# Keep quiet—how stress regulates hair follicle stem cells

**DOI:** 10.1038/s41392-021-00772-4

**Published:** 2021-10-08

**Authors:** Sven R. Quist, Jennifer Quist

**Affiliations:** 1 Department of Dermatology, University Hospital, Otto-von-Guericke University, Magdeburg, Germany; 2 Dermatology Clinic, Helix Medical Excellence Center, Mainz, Germany

**Keywords:** Skin stem cells, Skin stem cells

In a recent paper published in *Nature*, Choi et al.^1^ demonstrated that corticosterone, the mouse equivalent to human cortisol, controls the activity of hair follicle stem cell (HFCS) quiescence by regulating the gene expression of Gas6.

**Fig. 1:**
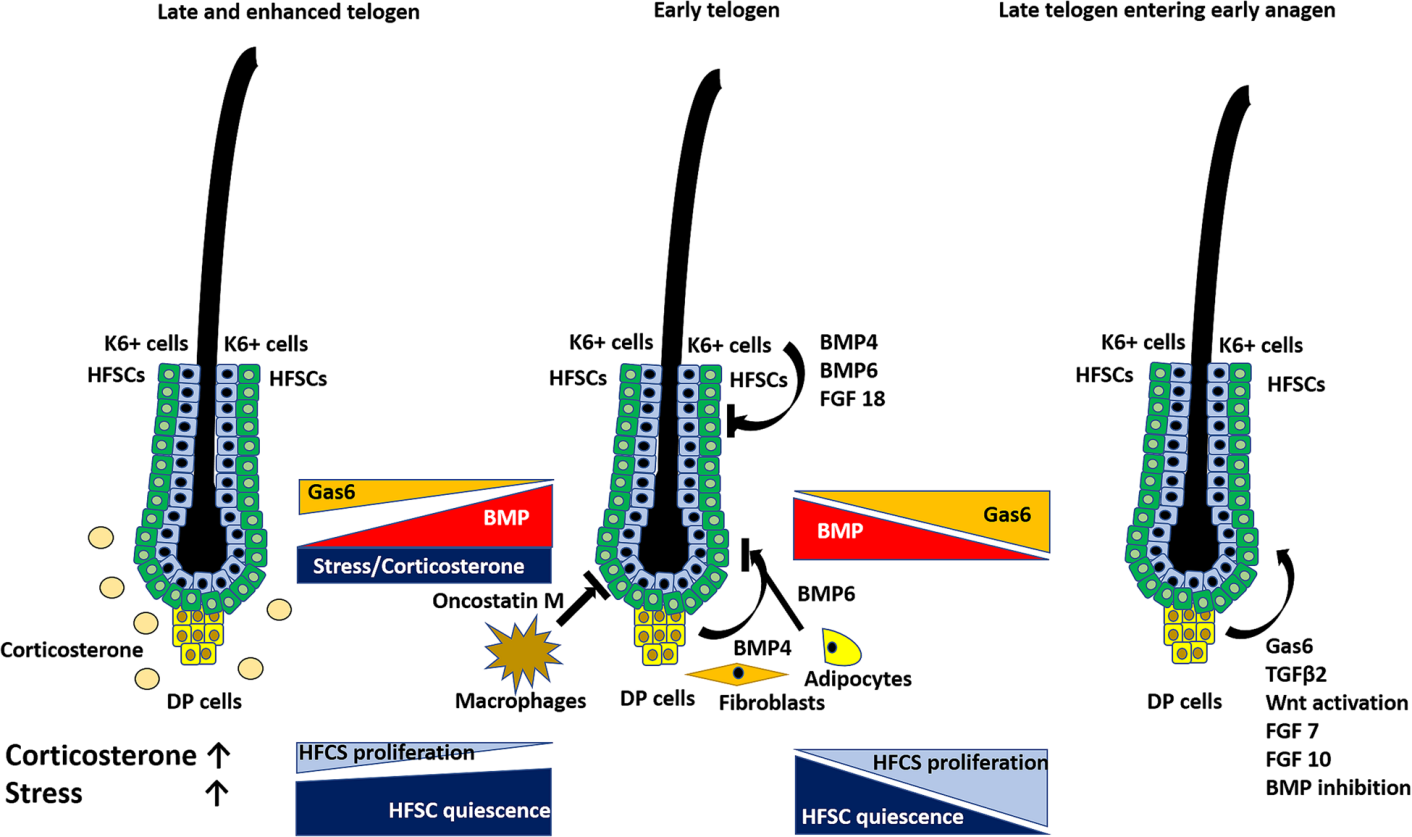
Regulation of hair follicle stem cell activity under normal condition and during enhanced corticosterone/stress. HFSC quiescence is regulated by the bulge niche environment. In early telogen, HFCSs remain quiescent through BMP6 and fibroblast growth factor 18, which are secreted from keratin 6+ cells of the inner bulge and BMP4 from DP cells, BMP2 and BMP4 from surrounding fibroblasts and adipocytes and oncostatin M from TREM2+ macrophages. In late telogen, Gas6, BMP inhibitors and TGF-β-2, FGF 7 and 10 activate Wnt signalling, leading to early anagen. High levels of corticosterone/stress suppress Gas6 and prevent the transition from late telogen to anagen prolonging late telogen

Mammalian stem cells have the unique ability to repair, generate and maintain tissues throughout their lifetime. Stem cells under autonomous and non-autonomous regulators are located in specific niches where this regulation can be synchronized.^2^ The hair follicle is a highly interactively regulated mini-organ that enables the study of stem cell functions. During hair formation, hair follicles undergo orchestrated and dynamic cycles of growth (anagen), regression (catagen) and quiescence (telogen). The niche of HFSCs is known as the bulge region where quiescent HFSCs are localized. HFSCs are transiently activated, and proliferate to form the hair germ. Matrix cells of the hair bulb proliferate and differentiate, elongating the hair shaft in the growth (anagen) phase. In the regression (catagen), the hair bulb and the lower part of the hair follicle regress leading to the telogen (resting) phase where bulge cells remain inactivated, and HFCSs are quiescent.^3^ In the telogen phase, under normal conditions, HFSC quiescence is regulated by cell intrinsic as well as extrinsic signals from the bulge niche environment. This is precisely orchestrated by Wnt and bone morphogenetic protein (BMP) signalling. In early telogen, HFCSs are silenced through BMPs, including BMP6 and fibroblast growth factor 18, secreted from Keratin 6-positive cells of the inner bulge, BMP4 from dermal papilla cells that mantain a low Wnt low niche environment and BMP2 and BMP4 from surrounding fibroblasts and adipocytes.^3^ Transcriptional factors such as Nfatc1 or Foxp1 are important intrinsic regulators of HFSC quiesence that control BMP signalling.^2^ TREM2-positive macrophages in surrounding tissues secrete oncostatin M^4^ which activates JAK-STAT5 signalling to mantain HFSC quiescence. During the early telogen phase, the HFSC niche environment is BMP-high and Wnt-low to mantain HFSC in quiescence. Dermal papilla (DP) cells play a critical regulatory role as they secrete various mediators in a paracrine manner either to keep HFSC inactivity or to enter the early anagen phase by secreting BMP inhibitors and transforming growth factor-beta-2, Fibroblast growth factor 7 and 10 activate Wnt signalling leading to the early anagen phase again (Fig. 1).

Chronic stress can result in continuous hair loss. This leads to telogen effluvium where hair follicles enter a resting phase followed by considerable hair loss. Even worse, it can also result in an autoimmune condition called alopecia areata where hairs are lost due to an attack of the immune system,^5^ resulting in complete hait loss of the hair of the scalp or body. It is thought that, under stress, the hair follicle is under a micro-inflamed environment^5^ and may need more of its essential nutrients such as proteins, vitamins, iron or zinc. Choi and coworkers showed that chronic stress has an even more profound impact on hair growth by governing HFSCs, keeping them in a quiescent state by changing the microenvironment. In various mouse models, by adenovirus-associated injection with activating gene expression and cell culture experiments, they studied the effect of corticosterone on the activity of HFSCs. Hairs of mice that underwent surgical removal of the adrenal glands (adrenalectomy) remained proliferative, their hair shafts and hair follicles became longer, and their hair follicles entered anagen phases more synchronized with longer cycles compared with the results obtained in mice with intact glands. To study the direct effect of corticosterone on hair follicle growth, mice were fed oral corticosterone. Hair follicles prolonged their telogen phase in contrast to that observed in mice without adrenal glands. During ageing, mice showed increased levels of corticosterone and long telogen phases could be reversed by re-entry in the anagen phase by adrenalectomy, even in old mice. Anagen entry remained intact throughout life. In a mouse model of growth receptor gene deletion, they revealed that the dermal papilla fibroblasts surrounding the hair follicle play a crucial role in connecting the corticosterone effects with the prolonged resting phases. By comparing the different mouse models, DP cells revealed a candidate gene, Growth Arrest Specific Protein 6 (Gas6), as a regulator of hair growth that is stress sensitive. In DP cells, Gas6 encodes a gamma-carboxy glutamic acid-containing secreted protein that predominantly binds to AXL, a member of the TAM (TYRO3, AXL and MERTK)-family of receptor tyrosine kinases and can be suppressed by corticosterone. Interestingly, recombinant Gas6 was also able to activate hair growth when supplemented in vitro with isolated HFSCs and in vivo via adenovirus-associated injection.

High levels of BMPs secreted from fibroblasts, and dermal adipocytes can promote HFSC quiescence in early (refractory) telogen. Since the effects of corticosterone remain constant, Choi and coworkers observed that Gas6 expression was opposite to BMP signalling. Gas6 expression in the dermal papilla was low in early telogen and high in late telegon. BMP suppressed the expression of Gas6, and when Noggin, a secreted BMP inhibitor, was overexpressed, increased levels of Gas6 were observed. Corticosterone (a systemic hormone) and BMP (a niche signal) represent two upstream signals that suppress Gas6 levels in DP. Restoration of Gas6 expression via adenovirus-associated injection was sufficient to promote HFSC activation even in a high-corticosterone environment, suggests that corticosterone regulates HFSC activity by inhibiting Gas6 expression in dermal papilla cells.

This work is of interest because the orchestrated signalling process of mammalian HFSCs during maintenance and repair, already known to be quite complex, has been a primary research focus. Nevertheless, the influence of external stimuli such as corticoids (in mice, corticosterone is the major effector of corticoids), on the regulation of HFSCs, has not yet been well characterized. Chronic stress, as well as long-term corticoid therapy, has many undesirable side effects. It seems to directly impact the regulation of hair follicle stem cells via non-autonomous, paracrine effects keeping those cells in both, a prolonged quiescent state and prolonged telogen phase. This results in a noticeable stop of hair growth and potential subsequent telogen hair loss, which is a further stressor for patients.
